# MLXIPL promotes the migration, invasion, and glycolysis of hepatocellular carcinoma cells by phosphorylation of mTOR

**DOI:** 10.1186/s12885-023-10652-5

**Published:** 2023-02-21

**Authors:** Xiaowei Chang, Chang Tian, Yuanyuan Jia, Yu Cai, Pu Yan

**Affiliations:** 1 Department of General Surgery, The First Affiliated Hospital of Xi’an Medical University, No. 48, Fenghao West Road, Lianhu District, 710077 Xi’an, Shaanxi China; 2 Department of Clinical Laboratory, The First Affiliated Hospital of Xi’an Medical University, Xi’an, Shaanxi China; 3 Department of Faculty Development and Teaching Evaluation Office, The First Affiliated Hospital of Xi’an Medical University, Xi’an, Shaanxi China

**Keywords:** Hepatocellular carcinoma, MLXIPL, mTOR, Glycolysis, Invasion, Migration

## Abstract

**Background:**

Hepatocellular carcinoma (HCC) is associated with a high occurrence, mortality, and poor prognosis. MLX interacting protein like (MLXIPL) is an important regulator of glucolipid metabolism and is involved in tumor progression. We aimed to clarify the role of MLXIPL in HCC and its underlying mechanisms.

**Methods:**

The level of MLXIPL was predicted using bioinformatic analysis and verified using quantitative real-time PCR (qPCR), immunohistochemical analysis, and western blot. We assessed the effects of MLXIPL on biological behaviors using the cell counting kit-8, colony formation, and Transwell assay. Glycolysis was evaluated using the Seahorse method. The interaction between MLXIPL and mechanistic target of rapamycin kinase (mTOR) was confirmed using RNA immunoprecipitation and co-immunoprecipitation. mTOR expression was detected in HCC cells using qPCR, immunofluorescence analysis, and western blot.

**Results:**

The results showed that MLXIPL levels were elevated in both HCC tissues and HCC cell lines. Knockdown of MLXIPL impeded HCC cell growth, invasion, migration, and glycolysis. Moreover, MLXIPL combined with mTOR to induce phosphorylation of mTOR. Activated mTOR abrogated the effects on cellular processes induced by MLXIPL.

**Conclusion:**

MLXIPL promoted the malignant progression of HCC by activating phosphorylation of mTOR, suggesting an important role of the combination of MLXIPL and mTOR in HCC.

**Supplementary Information:**

The online version contains supplementary material available at 10.1186/s12885-023-10652-5.

## Introduction

Liver cancer, a multilayered heterogeneous tumor, is the fourth leading cause of cancer-associated deaths [1, 2]. The most common pathological type of primary liver cancer is hepatocellular carcinoma (HCC). Although surgical resection, liver transplantation and radiofrequency ablation provide multiple approaches for the treatment of HCC, most patients are at an advanced stage, and their primary treatment of choice is chemotherapy [3]. Moreover, due to the high recurrence and metastasis of HCC, the prognosis of HCC is extremely poor [4]. The molecular mechanisms of HCC oncogenesis and development are very complex, and include changes in the tumor microenvironment, single nucleotide polymorphisms (SNP), somatic gene mutations, and abnormal signaling pathway regulation [5, 6]. Thus, further understanding of the pathogenesis of HCC is urgently required.

Glycolysis is the preferred method of energy metabolism for tumor cells, and targeting glycolysis may provide new opportunities for tumor therapy [7]. Aerobic glycolysis in tumor cells is linked to uncontrolled cell proliferation. Glycolysis provides NADPH and ATP, which are also present under anoxic conditions, and continuously provide energy to cancer cells [8]. Aerobic glycolysis is a classic indicator of HCC and is responsible for the proliferation, immune escape, metastasis, and angiogenesis of HCC cells [9]. Therefore, clarifying the mechanisms of glycolysis will contribute to the development of new approaches for HCC treatment.

MLX interacting protein like (MLXIPL), also known as ChREBP, is a transcription factor regulating lipogenesis in liver tissues [10]. It can be activated by carbohydrate metabolites and counter-activate the glucose metabolic process by regulating glycolysis during the circulation of sugars [11]. MLXIPL is highly expressed in human metabolic tissues, including the liver, islets, intestinal, and renal tissues, and is expressed at low levels in other tissues such as skeletal muscle [12]. Dysregulation of MLXIPL is associated with coronary artery disease, fatty liver disease, and malignancy [13–15]. MLXIPL is significantly associated with the prognosis of HCC and promotes cell proliferation and glycolysis [16]. However, the mechanisms underlying MLXIPL remain unclear.

In this study, we explored the effects of MLXIPL on cellular processes in HCC, including proliferation, metastasis, and glycolysis. Additionally, we revealed the mechanisms of underlying MLXIPL. We found that MLXIPL knockdown inhibited malignant cell phenotypes by regulating mTOR. The findings suggest an important role for the combination of MLXIPL and the mTOR axis in HCC.

## Materials and methods

### Bioinformatic analysis

MLXIPL expression in patients with HCC (n = 374) and normal healthy (n = 50) was predicted using the starBase online database (https://starbase.sysu.edu.cn/index.php). MLXIPL associated functional partners were predicted using the STRING database (https://cn.string-db.org/).

### Tissue samples

All operations involving human tissues were approved by the ethics committee of the First Affiliated Hospital of Xi’an Medical University, and tumor tissues and pericarcinoma liver tissues (PCLT) were obtained from patients with HCC during surgery (n = 56). All tissues were stored at -80 °C until further use. All the participants provided written informed consent. The clinical characteristics related to low or high expression of MLXIPL were listed in Table 1.

**Table 1:** Clinicopathologic characteristics of study subjects

Clinicopathologic characteristics	*n*	Low	High	*p*-value	
**Age (years)**				0.8297	
< 65	27	11	16		
≥ 65	29	11	18		
**Sex**				0.2249	
Male	38	17	21		
Female	18	5	13		
**AJCC stage**				0.0163*	
I + II	35	18	17		
III + IV	21	4	17		
**T stage**				0.0482*	
T1 + T2	29	15	14		
T3 + T4	27	7	20		
**Differentiation**				0.0354*	
High	31	16	15		
Low	25	6	19		
**Tumor size**				0.0600	
≥50 mm	22	12	10		
≥ 50 mm	34	10	24		

### Immunohistochemical (IHC) analysis

Paired HCC and PCLT tissues (n = 5) were randomly selected and embedded in paraffin and cut into sections. The tissue sections were deparaffinized and rehydrated. Antigen retrieval was performed using a 0.01 M citric acid buffer. After washing with PBS, the sections were blocked with normal serum. Sections were incubated with anti-MLXIPL (ab92809, 1/500, Abcam, Cambridge, MA, USA) at 4 °C overnight and with a secondary antibody (ab6721, 1/1000, Abcam) at 37 °C for 30 min. After staining with DAB solution, the results were observed under a microscope (Olympus, Tokyo, Japan). IHC score was calculated as previously described [17]. Briefly, immunoreaction intensity was defined as follows: negative: 0, weak: 1, moderate: 2, strong: 3. The staining extent score was defined as 0-100%. The score was calculated as multiply immunoreaction intensity by by the staining extent score. The results were negative (0), weak (0 ~ 1), moderate (1 ~ 1.5), and strong (1.5 ~ 3).

### Cell culture

HCC cell lines (Hep3B, Huh7, Focus, HA22T) and THLE3 normal liver cells were acquired from the ATCC. All cells were incubated at DMEM (Hyclone) supplemented with 10% FBS (Hyclone) at 37 °C in a 5% CO_2_ atmosphere.

### Cell transfection

Hep3B and Huh7 cells were inoculated into six-well plates and cultured for 24 h. Then, cells were transfected with si-MLXIPL and si-MLXIPL (GenePharma, Shanghai, China) using Lipofectamine 3000 (Invitrogen). Six hours later, the culture medium was replaced with a complete medium. 24 h after transfection, the cells were collected for further studies.

### Cell viability detection

Cell Counting kit-8 (Dojindo, Kumamoto, Japan) was used to assess cell viability. Transfected cells were seeded in 96-well plates and incubated at 37 °C for 24 h. Subsequently, 10 µL CCK-8 solution was incubated with the cells for 2 h. Absorbance was detected using a microplate reader (Bio-Rad, Hercules, CA, USA) at 450 nm.

### Colony formation detection

Transfected cells were plated in six-well plates and incubated at 37 °C with 5% CO_2_. After 2 weeks, the cells were immobilized with 4% paraformaldehyde (PFA) and stained with crystal violet. The stained cells were observed.

Cell migration and invasion analysis.

Chambers without Matrigel and Matrigel-coated chambers (BD Biosciences, San Jose, CA) were used for determination of cell migration and invasion, respectively. Transfected cells were added to the upper chambers, and the medium was added to the lower chambers. After 24 h, the migrated and invasive cells were immobilized using 4% paraformaldehyde and stained with crystal violet. The stained cells were visualized under a microscope (Olympus). The numbers of migratory and invading cells were quantified in five random fields.

### Measurement of glycolysis

Oxygen consumption rate (OCR) and extracellular acidification rate (ECAR) were measured as previously showed [18]. Briefly, Hep3B and Huh7 cells were seeded in 96-well plates. For OCR detection, the Seahorse automatically filled with oligomycin at 20 min, p-trifluoromethoxy carbonyl cyanide phenylhydrazone (FCCP) at 50 min, and antimycin A and rotenone at 80 min. For ECAR detection, glucose was added at 20 min, oligomycin was added at 50 min, and 2-deoxy-D-glucose (2-DG) was added at 80 min using Seahorse Autofill. OCR was detected using a MitoCheck Mitochondrial OCR assay kit (Cayman Chemical, Ann Arbor, MI). ECAR was detected using a Seahorse XF glycolysis stress test kit (Agilent, Beijing) from 0 to 110 min following the manufacturer’s protocol. The results were measured using Seahorse XF96 analyzer (Agilent).

### Quantitative real-time PCR (qPCR)

Total RNA was isolated from tissues and HCC cells using the RNA Simple Total RNA Kit (Tiangen, Beijing). After RNA purity and integrity testing, the cDNA was reverse transcribed using a Quant cDNA first strand synthesis kit (Tiangen). qPCR was performed using a FastFire SYBR Green qPCR PreMix kit (Tiangen) on a LightCycler Real-Time System (Roche, Basel, Switzerland). The expression (relative fold change) was quantified using the 2^−ΔΔCt^ method. β-actin was the internal control. The sequences of primers were: MLXIPL forward: 5’-GCAGTATCGACCCCACAC-3’ and reverse: 5’-TCCAGATGGCGTTGTTCA-3’; β-actin 5’-CTTAGTTGCGTTACACCCTTTCTTG-3’ and reverse: 5’-CTGTCACCTTCACCGTTCCAGTTT-3’.

### Western blot

HCC cells were harvested and lysed using RIPA reagent (Beyotime). After detecting the protein concentration using the BCA method, 30 µg of protein was electrophoresed on 10% SDS-PAGEs. After the proteins were transferred to PVDF membranes, they were blocked with 5% free-fat milk. The membranes were incubated with primary antibodies including anti-MLXIPL (ab92809, 1/1000), anti-mTOR (ab32028, 1/5000), anti-p-mTOR (phosphor S2448; ab131538, 1/1000) and anti-GAPDH (ab9485, 1/2500) at 4 °C for 12 h and subsequently incubated with secondary antibody (ab6721, 1/3000) at 25 °C for 2 h. All antibodies were purchased from Abcam. Protein bands were observed after development with ECL reagent (Sigma-Aldrich, St. Louis, MO).

### Immunofluorescence (IF) analysis

Transfected Hep3B and Huh7 cells were immobilized with 4% PFA, permeabilized with Triton X-100, and blocked using normal goat serum. The cells were incubated with anti-p-mTOR (phosphor S2448; ab131538, 1/100; Abcam) at 4 °C for 12 h and incubated with a secondary antibody (ab150077, 1/200) at 37 °C for 1 h. The signals were visualized using a confocal laser scanning microscope (Olympus).

### RNA immunoprecipitation (RIP) analysis

RIP was performed as previously described [18]. An EZ-Magna RIP kit (Merck Millipore, Billerica, MA, USA) was used. Hep3B and Huh7 cells were lysed using RIP lysis for 10 min. Cell lysates were incubated with magnetic beads containing protein A/G conjugated with MLXIPL antibody or anti-IgG at 4 °C overnight. The enrichment of mTOR was examined by qPCR.

### Co-immunoprecipitation (Co-IP) assay

Co-IP was conducted as previously described [19]. MLXIPL transfected cells were lysed using a lysis buffer and centrifuged at 12,000×g for 10 min. The supernatant was incubated with Protein A/G PLUS-Agarose (Santa Cruz Biotechnology, Santa Cruz, CA, USA) and anti-mTOR at 4 °C overnight. Following washing, the immunoprecipitants were boiled in 2× sodium dodecyl sulfate (SDS) loading buffer. The expression of mTOR was measured using western blot. The antibodies using in Co-IP-western blotting were anti-MLXIPL (sc-515,922, 1/100, Santa Cruz Biotechnology) and anti-mTOR (ab2732, 1/100, Abcam).

### Statistical analyses

Data are presented as the mean ± standard deviation and were analyzed using GraphPad Prism 8 software (GraphPad, La Jolla, CA, USA). Differences were assessed using Student’s t-test or one way ANOVA. Statistical significance was set at p < 0.05.

## Results

### MLXIPL is upregulated in HCC tissues and cells

**Fig. 1:**
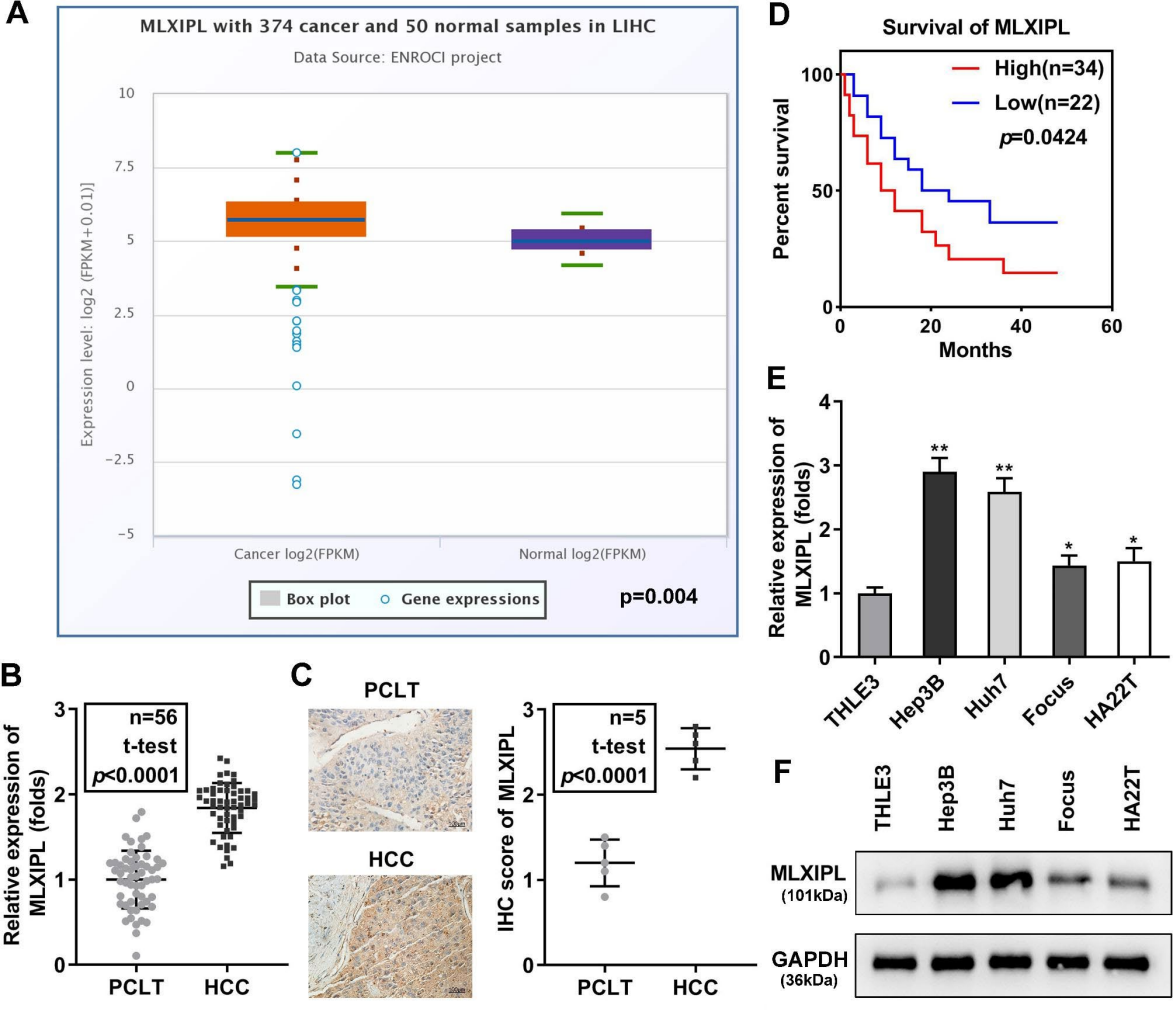
MLXIPL is upregulated in HCC. (A) MLXIPL expression in tumor (n = 374) and normal control (n = 50) was predicted using the starBase database. MLXIPL in PCLT tissues and HCC tissues (n = 56) was measured using (B) qPCR. (C) The MLXIPL level in PCLT and HCC tissues were assessed by IHC assay and IHC score was quantified. (D) The survival rate of HCC patients with a high or low expression of MLXIPL. MLXIPL in THLE3, Hep3B, Huh7, Focus, and HA22T cells was measured using (E) qPCR and (F) western blot. **P < 0.01. *P < 0.05

We studied metabolism-related MLXIPL gene in HCC and we first detect its expression. Bioinformatics predicted that the levels of MLXIPL were higher in patients with HCC than in normal subjects (p = 0.004; Fig. 1A).

To confirm this prediction, we collected 56 paired HCC tissues and PCLT tissues for this study. The qPCR results illustrated that MLXIPL levels were significantly higher in HCC tissues than in PCLT tissues (Fig. 1B). The data from the IHC assay also showed that MLXIPL was elevated in the tumor tissues (Fig. 1C). Patients with high levels of MLXIPL had shorter survival than those with low MLXIPL expression (Fig. 1D). Moreover, MLXIPL expression was markedly increased in Hep3B, Huh7, Focus, and HA22T cells, compared with THLE3 cells, especially in Hep3B and Huh7 cells (Fig. 1E and F). The abnormal expression of MLXIPL was related to tumor stage and differentiation but was not related to age, sex, or tumor size (Table 1).

### Knockdown of MLXIPL suppresses cell proliferation, migration, invasion, and glycolysis

**Fig. 2:**
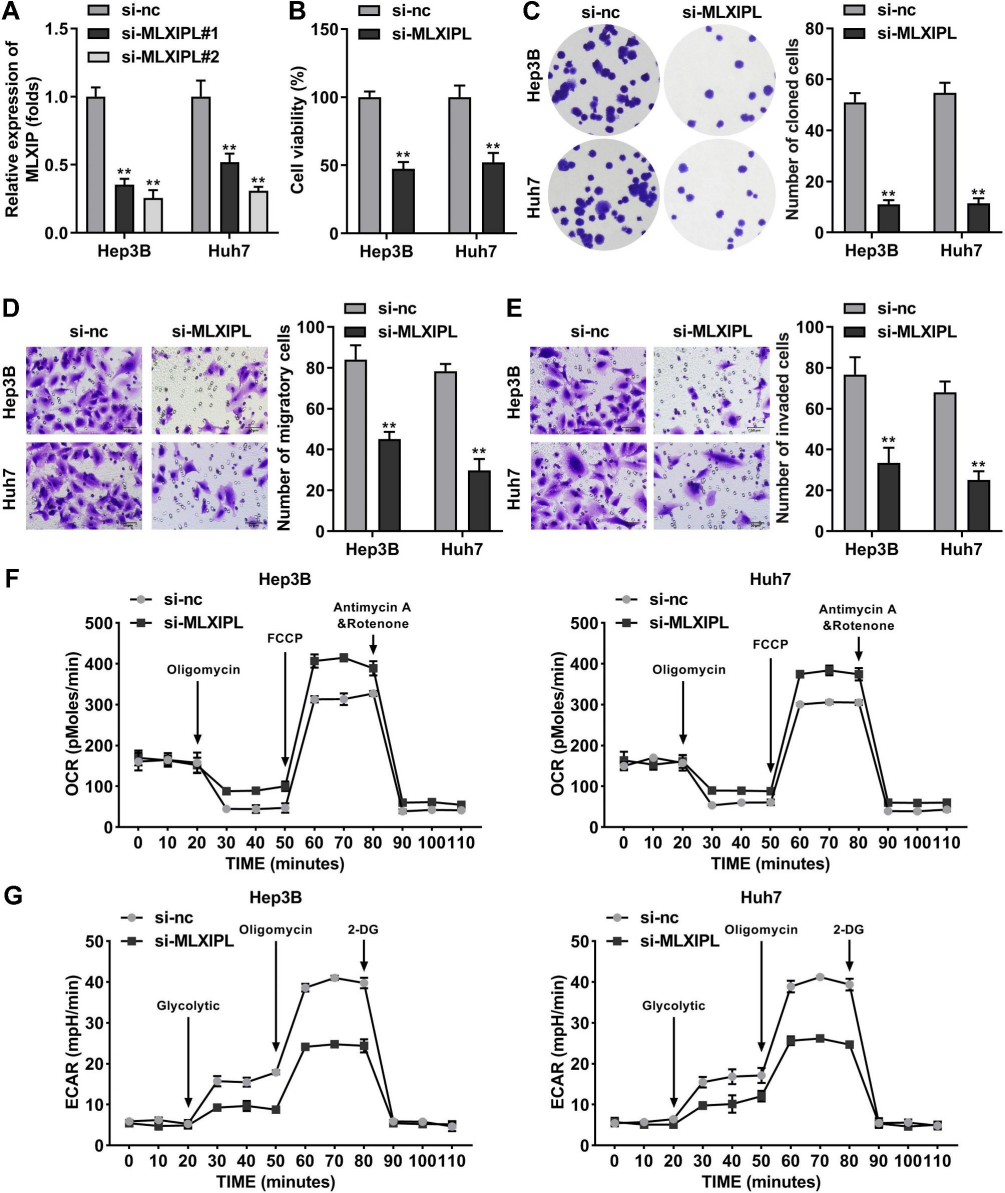
Knockdown of MLXIPL suppresses cell proliferation, migration, invasion, and glycolysis. (A) MLXIPL expression was detected using qPCR in si-MLXIPL #1, si-MLXIPL #2, and si-NC transfected cell. Cell proliferation was evaluated by detecting (B) cell viability using CCK-8 and (C) cell colonies using colony formation assay (magnification 10×). Transwell assay measured (D) cell migration and (E) cell invasion. (F) The OCR and (G) ECAR were analyzed as indicators of tumor cell glycolysis. **P < 0.01

To clarify the biological functions of MLXIPL, we first transfected Hep3B and Huh7 cells with si-MLXIPL and si-NC, respectively. MLXIPL expression was markedly downregulated in the MLXIPL vector-transfected cells (Fig. 2A). Loss of MLXIPL inhibited cell viability and colony formation (Fig. 2B and C). Subsequently, interference with MLXIPL inhibited cell migration and invasion (Fig. 2D and E). The ECAR to OCR ratios usually reflects the “Warburg” effect, indicating that they are glycolytic markers [20]. Activation of MLXIPL accelerates cell glycolysis in HCC [16]. Thus, we further demonstrated whether dysregulation of MLXIPL regulated glycolysis. The results showed that knockdown of MLXIPL increased OCR and decreased ECAR in both Hep3B and Huh7 cells, suggesting that MLXIPL promotes glycolysis (Fig. 2F and G).

### MLXIPL binds to mTOR to promote mTOR phosphorylation

**Fig. 3:**
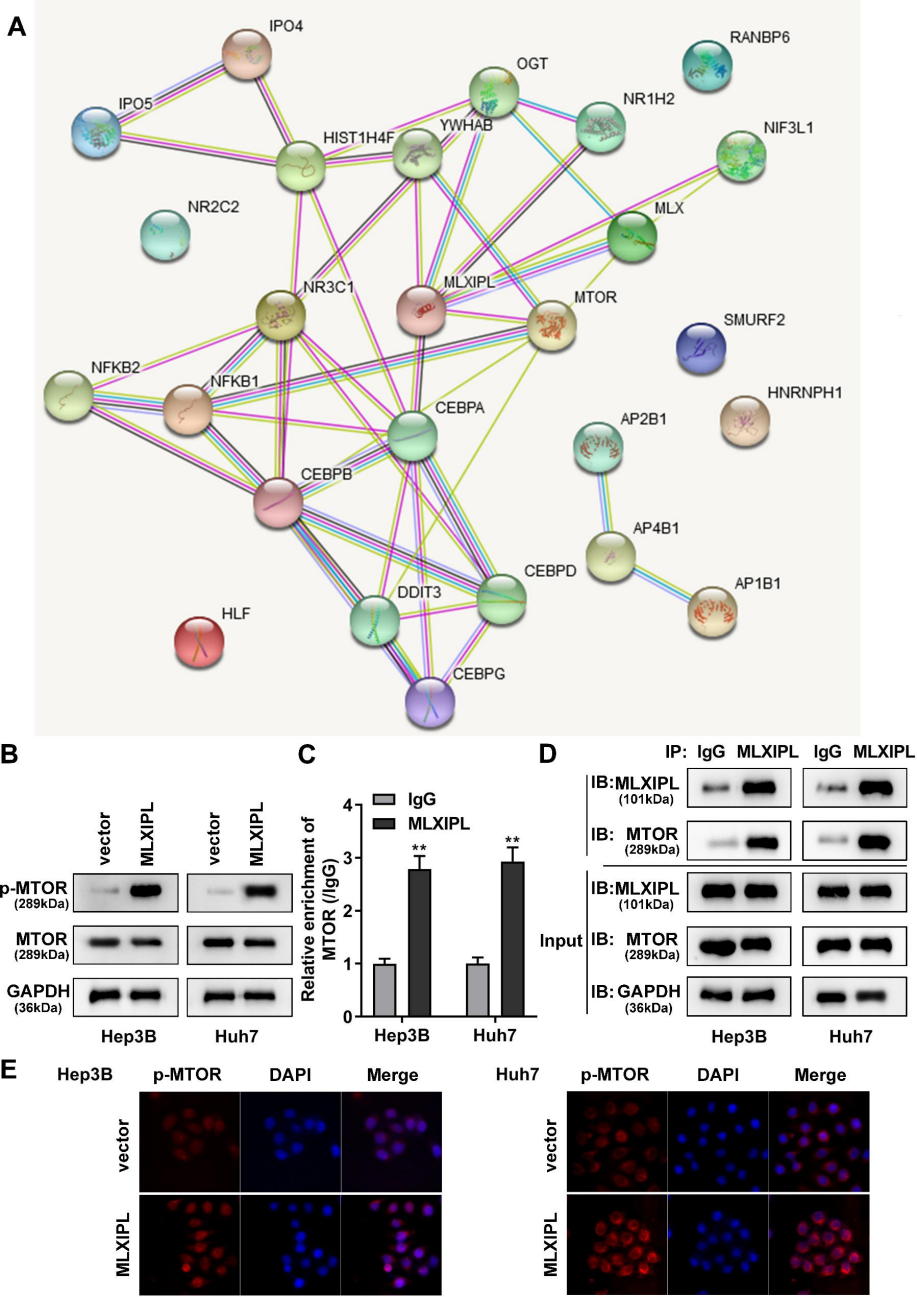
MLXIPL directly binds to mTOR. (A) The STRING database was used to predict MLXIPL associated functional partners. (B) The levels of mTOR and p-mTOR were examined using western blot. The combination of MLXIPL and mTOR was analyzed using (C) RIP assay and (D) Co-IP assay. (E) The protein levels of p-mTOR were observed using IF staining assay. **P < 0.01

To explore the underlying mechanisms, we used the STRING database to predict MLXIPL related proteins. The data showed that MLXIPL expression was closely related to mTOR (Fig. 3A). Overexpression of MLXIPL induced the upregulation of phosphorylated mTOR, but did not influence mTOR levels (Fig. 3B). Then, the combination of MLXIPL and mTOR was confirmed using RIP and Co-IP assay. The results showed that MLXIPL could bind to mTOR at mRNA and protein levels (Fig. 3C and D). The results of IF indicated that overexpression of MLXIPL increased the protein levels of p-mTOR in Hep3B and Huh7 cells (Fig. 3E).

### MLXIPL overexpression promotes malignant phenotypes of HCC cells by regulating mTOR

**Fig. 4:**
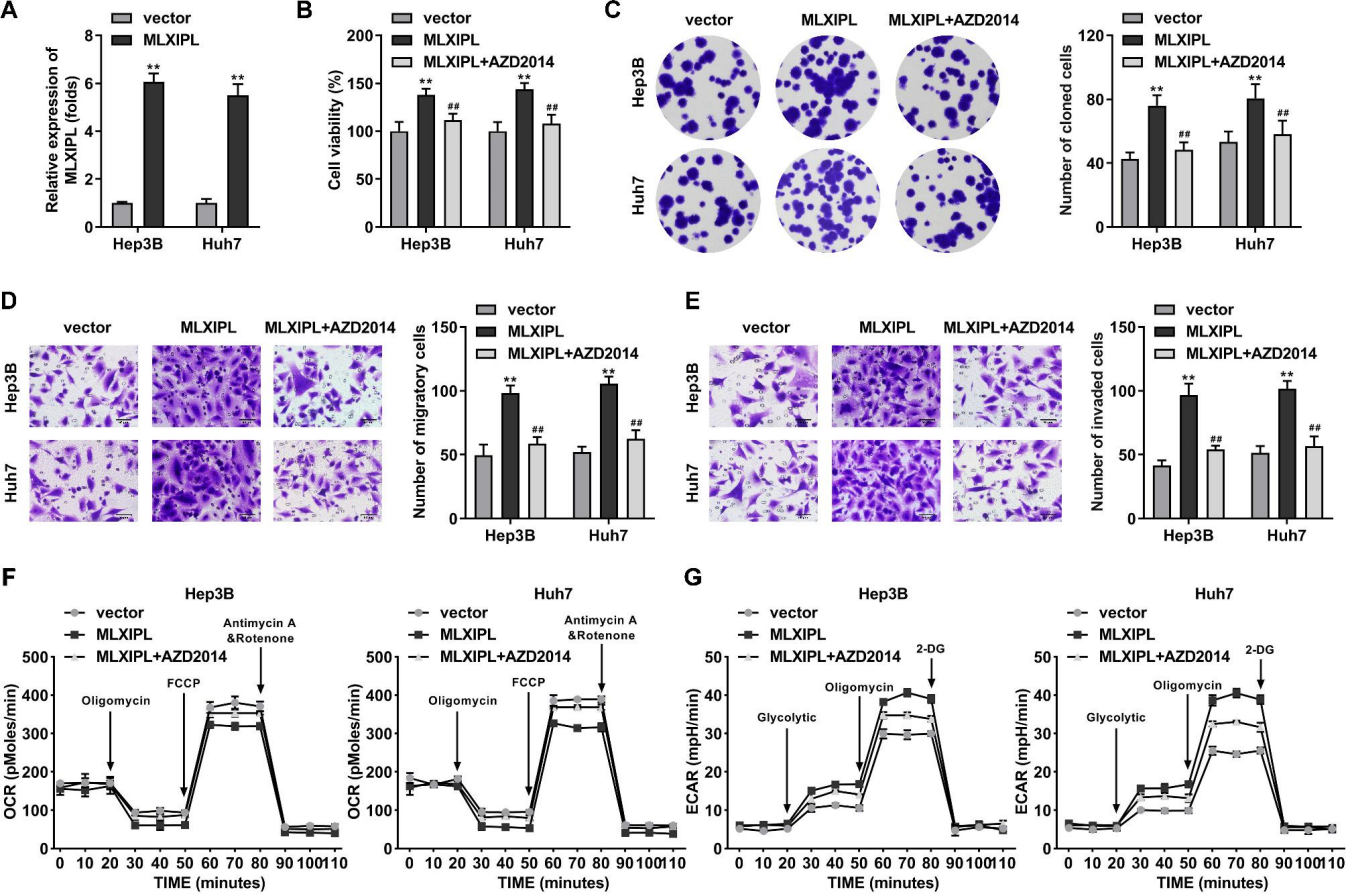
MLXIPL overexpression promotes malignant phenotypes of HCC cells by regulating mTOR. (A) The protein level of mTOR in mTOR stimulated Hep3B and Huh7 cells. (B) Cell viability was detected using CCK-8 assay. (C) Cell colonies were measured using colony formation assay (magnification 10×). (D) Cell migration and (E) invasion was examined by Transwell assay. (F) The OCR and (G) ECAR were analyzed as indicators of glycolysis of tumor cells. **P < 0.01. ##P < 0.01

For rescue experiments, the mTOR inhibitor AZD2014 was used to treat Hep3B and Huh7 cells, and mTOR expression was found to be significantly increased (Fig. 4A). Overexpression of MLXIPL facilitated cell proliferation, whereas AZD2014 abrogated proliferation (Fig. 4B and C). AZD2014 counteracted the promotion of MLXIPL-induced cell migration and invasion (Fig. 4D and E). MLXIPL increased OCR and reduced ECAR, whereas AZD2014 reversed the effects on OCR and ECAR induced by MLXIPL (Fig. 4F and G).

## Discussion

Glycolysis is first found in HCC and mediates tumor cell proliferation, metastasis, immune response, and drug resistance [9]. During glycolysis, ATP is produced and supplies more than 50% of the energy required by tumor cells. Lactic acid is produced and promotes tumor invasion and migration [21, 22]. Invasion and migration are important prerequisites for the distal metastasis of cancer cells [23] and are important factors leading to high cancer mortality and poor prognosis of HCC. Therefore, blocking the glycolysis, invasion and migration of HCC cells can help attenuate the progression of the disease and provide more opportunities for curing HCC.

MLXIPL is a glucose response factor that mediates glycolysis and lipogenesis. This leads to tumor cell proliferation by accelerating aerobic glycolysis [15]. Knockdown of MLXIPL impedes colon cancer cell glycolytic and lipogenic pathways, thereby inhibiting cell proliferation and blocking the cell cycle [24]. Dong et al. reported that MLXIPL is downregulated in HCC, and promotes proliferation, and glycolysis, and inhibits apoptosis [16]. Lei et al. also reported that the protein expression of MLXIPL is positively correlated with the degree of malignancy of HCC [25]. Based on these studies, we demonstrated that MLXIPL expression was decreased in HCC. Silencing of MLXIPL inhibited the proliferation, migration, invasion, and glycolysis of HCC cells. These findings suggest that MLXIPL has tumor promoting effects in HCC. Although the results of this study are partially consistent with the findings of Dong et al., we studied the biological behaviors of uncontaminated Hep3B and Huh7 cell lines, which are more convincing than the SMMC-7721 and HepG2 cell lines studied by Dong et al. Moreover, we investigated the regulation of MLXIPL on the invasion and migration of HCC cells, providing a more comprehensive theoretical basis for the role of MLXIPL in HCC.

mTOR is a serine/threonine kinase assembled into mTOR complex 1/2, which is commonly dysregulated in malignancies and causes somatic mutations [26]. mTOR regulates protein synthesis, metabolism, signal transduction, and cell growth by catalyzing S6K1, PKC, GF-IR and other factors. In human cancers, mTOR activation facilitates tumor cell proliferation and migration [27]. The mTOR related PI3K/Akt/mTOR signaling pathway provides a theoretical basis for mTOR-targeted therapy in cancer [28]. In this study, we found that mTOR expression was closely correlated with MLXIPL. MLXIPL activated mTOR phosphorylation. Overexpression of MLXIPL facilitates the malignant biological behaviors of HCC cells, whereas inactivation of mTOR using mTOR inhibitor abrogated the effects on cell growth, metastasis, and glycolysis induced by MLXIPL. These findings reveal a novel underlying mechanism of MLXIPL, suggesting that MLXIPL promoted the progression of HCC via inactivating mTOR. Accumulating evidence has shown that mTOR regulates the transcriptional activity and expression of MLXIPL [29, 30]. In contrast to previous studies, we found for the first time that MLXIPL could bind to mTOR and mediates its phosphorylation. This indicated that there is a positive feedback mechanism between MLXIPL and mTOR that can regulate each other.

In conclusion, MLXIPL acts as a tumor promoter in HCC. The loss of MLXIPL suppresses cell proliferation, invasion, migration, and glycolysis in HCC by inactivating mTOR phosphorylation. These data suggest that MLXIPL has the potential to treat HCC.

## Electronic supplementary material

Below is the link to the electronic supplementary material.

Supplementary Material 1

## Data Availability

The datasets used and analyzed during the current study are available from the corresponding author on reasonable request.
