# Supplementary figures and images for: MLXIPL promotes the migration, invasion, and glycolysis of hepatocellular carcinoma cells by phosphorylation of mTOR
Source: BMC Cancer. 2023 Feb 21;23:176. doi: 10.1186/s12885-023-10652-5 (PMC9945719; doi:10.1186/s12885-023-10652-5)

**Figure 1**

GAPDH


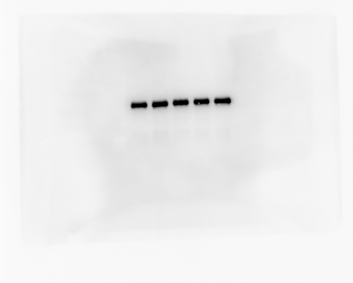


MLXIPL


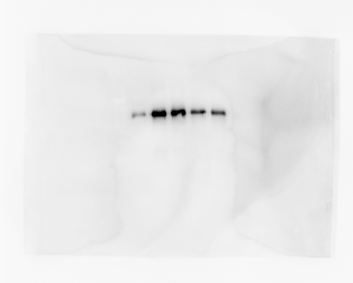


**Figure 3**

B-MTOR


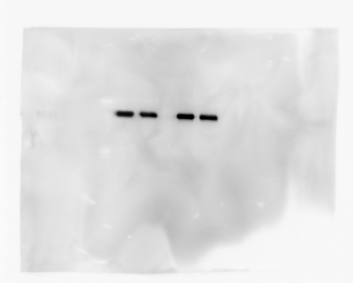


D-MTOR


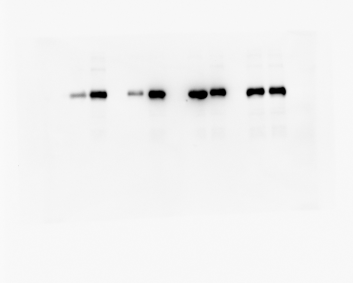


GAPDH


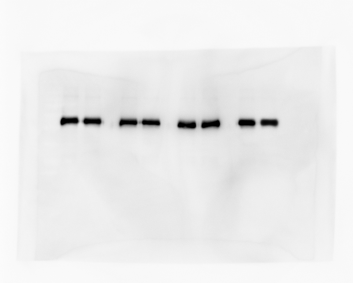


MLXIPL


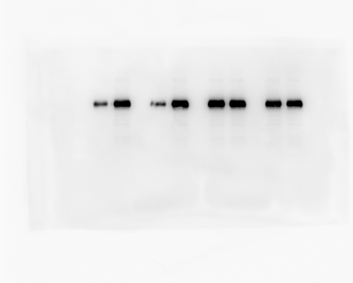


P-MTOR


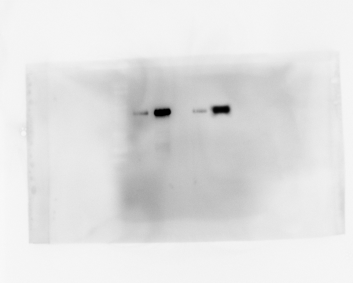

Supplement: Supplementary file 1 — Supplementary Material 1 [file 12885_2023_10652_MOESM1_ESM.docx]
